# The Effect of Renal Denervation on T Cells in Patients with Resistant Hypertension

**DOI:** 10.3390/ijms24032493

**Published:** 2023-01-27

**Authors:** Marta Kantauskaite, Oliver Vonend, Mina Yakoub, Philipp Heilmann, Andras Maifeld, Peter Minko, Lars Schimmöller, Gerald Antoch, Dominik N. Müller, Claudia Schmidt, Blanka Duvnjak, Ulf Zierhut, Sebastian A. Potthoff, Lars C. Rump, Johannes C. Fischer, Johannes Stegbauer

**Affiliations:** 1 Department of Nephrology, Medical Faculty, University Hospital Düsseldorf, Heinrich-Heine-University Düsseldorf, 40225 Düsseldorf, Germany; 2 Nierenzentrum Wiesbaden, 65191 Wiesbaden, Germany; 3 Charité-Universitätsmedizin Berlin, Corporate Member of Freie Universität Berlin and Humboldt-Universität zu Berlin, 10117 Berlin, Germany; 4 Experimental and Clinical Research Center, a Cooperation of Charité-Universitätsmedizin Berlin and Max Delbruck Center for Molecular Medicine, 13125 Berlin, Germany; 5 Department for Diagnostic and Interventional Radiology, Medical Faculty, University Hospital Düsseldorf, Heinrich-Heine-University Düsseldorf, 40225 Düsseldorf, Germany; 6 Institute of Transplantation Diagnostics and Cell Therapeutics, Medical Faculty, University Hospital Düsseldorf, Heinrich-Heine-University Düsseldorf, 40225 Düsseldorf, Germany

**Keywords:** sympathetic activity, renal denervation, resistant hypertension, immune response, T cells, lymphocytes, pro-inflammatory cytokines, inflammation

## Abstract

(1) Background: Sympathetic overactivity is a major contributor to resistant hypertension (RH). According to animal studies, sympathetic overactivity increases immune responses, thereby aggravating hypertension and cardiovascular outcomes. Renal denervation (RDN) reduces sympathetic nerve activity in RH. Here, we investigate the effect of RDN on T-cell signatures in RH. (2) Methods: Systemic inflammation and T-cell subsets were analyzed in 17 healthy individuals and 30 patients with RH at baseline and 6 months after RDN. (3) Results: The patients with RH demonstrated higher levels of pro-inflammatory cytokines and higher frequencies of CD4+ effector memory (T_EM_), CD4+ effector memory residential (T_EMRA_) and CD8+ central memory (T_CM_) cells than the controls. After RDN, systolic automated office blood pressure (BP) decreased by −17.6 ± 18.9 mmHg. Greater BP reductions were associated with higher CD4+ T_EM_ (r −0.421, *p* = 0.02) and CD8+ T_CM_ (r −0.424, *p* = 0.02) frequencies at baseline. The RDN responders, that is, the patients with ≥10mmHg systolic BP reduction, showed reduced pro-inflammatory cytokine levels, whereas the non-responders had unchanged inflammatory activity and higher CD8+ T_EMRA_ frequencies with increased cellular cytokine production. (4) Conclusions: The pro-inflammatory state of patients with RH is characterized by altered T-cell signatures, especially in non-responders. A detailed analysis of T cells might be useful in selecting patients for RDN.

## 1. Introduction

By affecting more than 1.5 billion people worldwide, hypertension is one of the most important global health concerns [1,2]. This is mostly because its complications, including stroke, heart failure and kidney disease, are the leading causes of cardiovascular morbidity and mortality [3,4]. Large randomized studies have shown the effectiveness of antihypertensive treatments [5]. However, despite the increasing awareness and the vast number of effective antihypertensive agents, hypertension control rates remain unsatisfactory. Typically, less than 50% of treated patients achieve their blood pressure targets [6,7]. A growing number of these cases are being called resistant hypertension (RH), which is defined as a blood pressure above 140/90mmHg despite the concurrent use of three antihypertensive agents, including a diuretic [8]. In this regard, patients with RH have the highest risk of cardiovascular events [9,10].

One of the cornerstones for the development and persistence of hypertension in RH is an increased activation of the sympathetic nervous system (SNS) [11,12]. In addition to SNS activation at a systemic level, renal sympathetic nerve activation seems to play an outstanding role in the pathogenesis of RH, as it leads to sodium and water retention, the activation of the renin–angiotensin system and vasoconstriction [13,14]. Therefore, the reduction of SNS activity at the renal level using catheter-based renal denervation (RDN) has become an interventional treatment approach for RH. By reducing sympathetic activity, a significant and longstanding blood pressure reduction in 60–70 % of cases can be achieved [15,16,17]. However, about one-third of patients do not respond to the procedure with respect to a significant blood pressure reduction. The reasons for non-response however are not well understood. More importantly, there are no reliable clinical or biochemical factors that can be applied to predict the success of the procedure [15,18].

For several years, there has been evidence supported mainly by experimental animal studies that SNS interacts with the immune system and modulates blood pressure, as well as the course of cardiovascular disease. It is known that primary and secondary lymphatic organs are innervated by SNS fibers [19]. Increased SNS activity, as in the case of RH, has been shown to drive immune cell functions and signatures into pro-inflammatory phenotypes and foster immune cell infiltration into target organs, such as the kidney or heart [20,21]. In this regard, norepinephrine released from sympathetic neurons alters the phenotypes and functions of monocytes, macrophages and T cells [19,22,23,24]. Specifically, CD8+ cells produce more pro-inflammatory cytokines and display enhanced survival upon increased SNS activity [22,24,25]. In fact, CD8+ T cells play a major role in the development of hypertension, as a lack of these immune cells is associated with protection against experimental hypertension in mice [26]. Unfortunately, despite a large body of experimental evidence, there are no human studies to date demonstrating that sympathetic overactivity in the presence of RH affects the T-cell response or that RDN can effectively modulate it.

In the present study, we aimed to describe the immune cell signatures of patients with RH and to compare them to those of healthy controls. Next, we aimed to determine whether these changes were modulated by RDN. Lastly, to improve patient selection for RDN, we sought to characterize the differences in immune cell phenotypes between the patients who responded to the RDN procedure and those who did not.

## 2. Results

### 2.1. Baseline Characteristics

A total of 30 patients with resistant hypertension (RH) with an average age of 61.1 ± 10.9 years were included in this study, and there was a slightly higher number of males (18 males and 12 females). According to 24-h blood pressure measurements, they had an average systolic blood pressure of 157 ± 15 mmHg and a diastolic blood pressure of 90 ± 11 mmHg before the renal denervation procedure (RDN). The antihypertensive regime consisted of 6 ± 1 drugs, and this regime was stable for a minimum of 4 weeks (Table 1). After inclusion in the study, all of the patients underwent RDN. In addition, we included 17 healthy controls, who presented with normal blood pressure and were 42.2 ± 11.3 years old.

### 2.2. Patients with Resistant Hypertension Display High Levels of Inflammatory Markers

**Figure 1:**
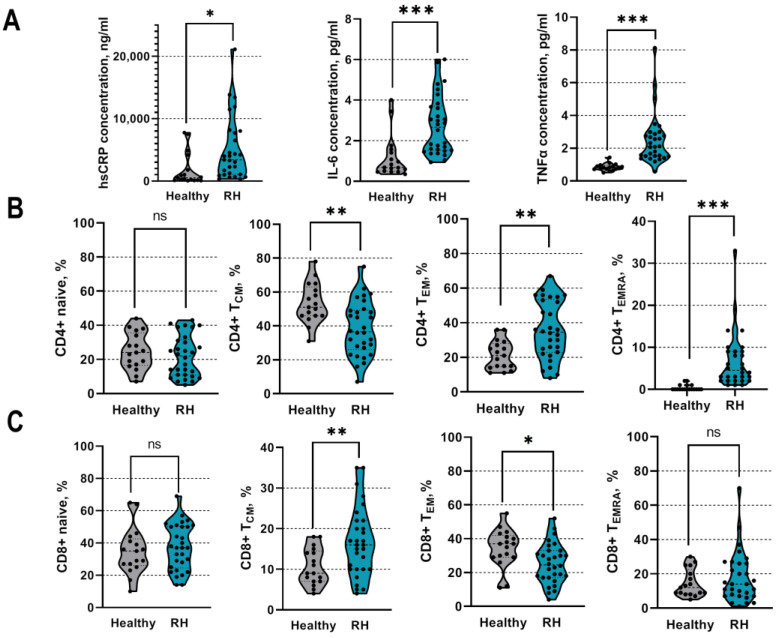
Comparison of inflammatory activity between patients with resistant hypertension and healthy controls. (**A**)—Concentrations of various pro-inflammatory cytokines and proteins among study groups at entry. (**B**,**C**)—The frequencies of different T-cell subsets among healthy individuals and patients with resistant hypertension at study entry. RH—resistant hypertension, hsCRP—high-sensitivity C-reactive protein, TNF-α—tumor necrosis factor alfa, IL-6—interleukin 6, CD4+—T helper cells, CD8+—T cytotoxic cells, T_CM_—central memory cells, T_EM_—effector memory cells, T_EMRA_—effector memory residential cells. *** represents significant difference between the groups with *p* < 0.001, ** *p* < 0.01, * *p* < 0.05, ns – not significant using unpaired *t*-test or Mann–Whitney test.

The patients with RH displayed significantly higher concentrations of high-sensitivity CRP than the healthy controls (hsCRP: 3691 (1126–7945) ng/mL vs. 513 (116–2993) ng/mL, *p* < 0.05). In addition, the patients with RH had higher levels of pro-inflammatory cytokines (TNF-α: 2.1 (1.5–2.8) pg/mL vs. 0.8 (0.7–0.9) pg/mL; IFN-γ: 2.7 (1.5–3.8) pg/mL vs. 0.7 (0.5–1.5) pg/mL, *p* < 0.001) than the healthy controls, as demonstrated in Figure 1A. Moreover, higher IL-6 levels were associated with higher diastolic blood pressure values among the patients with RH (r 0.404, *p* = 0.03). No relationship between cytokines and the systolic blood pressure values was observed.

### 2.3. Differences in T-Cell Subsets between Patients with Hypertension and Healthy Controls

Next, we analyzed the T-cell subsets and compared them between the healthy subjects and the patients with RH. The patients with RH presented higher frequencies of CD4+ effector memory T cells (T_EM_) (36 ± 16% vs. 21 ± 8.3%, *p* < 0.01) and CD4+ effector memory residential cells (T_EMRA_) than the control group (4.5 (2–9)% vs. 0.2 (0.1–0.6)%, *p* < 0.001, Figure 1B). In addition, the patients with RH had lower frequencies of CD4+ T_CM_ cells than the healthy controls (Figure 1B). As for CD8+ T cells, the patients with RH had a higher frequency of CD8+ T_CM_ cells (16 ± 8% vs. 11 ± 4%, *p* < 0.01) and a reduced fraction of CD8+ T_EM_ cells (25 ± 12% vs. 34 ± 11%, *p* < 0.05) compared to the healthy controls. There were no differences in other CD4+ or CD8+ subsets or in the frequencies of total CD4+ and CD8+ cells (Figure 1B,C).

### 2.4. Effect of RDN on Blood Pressure and Low-Grade Inflammation

**Figure 2:**
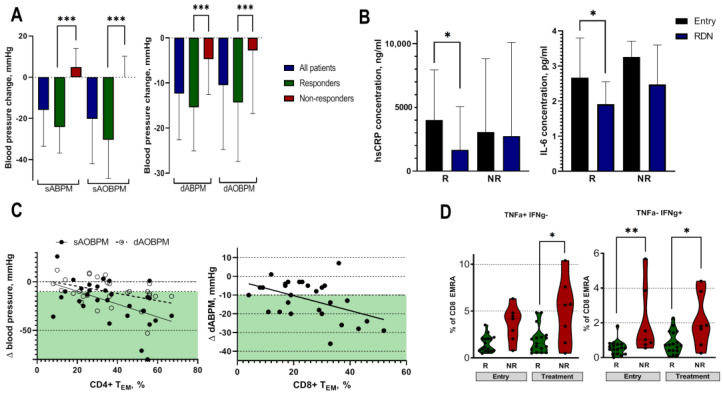
The effect of renal denervation procedure on blood pressure and inflammatory activity. (**A**)—Changes in ambulatory and automated office blood pressure among patients with resistant hypertension. Responders were defined by a systolic office blood pressure reduction of at least 10 mmHg following the renal denervation procedure. (**B**)—High-sensitivity CRP and IL-6 levels at baseline and 6 months following RDN among responders and non-responders. (**C**)—A negative correlation between CD4+ effector memory cell frequencies and automated office blood pressure was observed (r −0.421, *p* = 0.02, and r −0.413, *p* = 0.02, for systolic and diastolic blood pressure, respectively), as well as a negative correlation between CD8+ effector memory and diastolic ambulatory blood pressure (r −0.424, *p* = 0.02). Green colored area represents a blood pressure reduction ≥10mmHg. (**D**)—CD8+ T_EMRA_ cells positive for pro-inflammatory cytokines before and after the treatment among responders and non-responders. On the left, CD8+ T_EMRA_ cells positive for TNF-α and, on the right, the ones positive for IFN-γ. sABPM—systolic ambulatory blood pressure measurement, sAOBPM—systolic automated office blood pressure measurement, dABPM—diastolic ambulatory blood pressure measurement, dAOBPM—diastolic automated office blood pressure measurement, hsCRP—high-sensitivity C-reactive protein, IL-6—interleukin 6, RDN—renal denervation, R—responders, NR—non-responders, ∆—change, T_EM_—effector memory cells, T_EMRA_—effector memory residential cells, TNF-α—tumor necrosis factor alfa, IFN-γ—interferon gamma. *** represents significant difference between the groups with *p* < 0.001, ** *p* < 0.01, * *p* < 0.05 using unpaired *t*-test or Mann–Whitney test.

Six months after RDN, there was an average decrease in systolic automated office blood pressure of −17.6 ± 18.9 mmHg, whereas diastolic ambulatory blood pressure decreased by −8.7 ± 11.9 mmHg. Twenty patients (67%) achieved at least a 10 mmHg reduction in their systolic office blood pressure and were assigned to the responder group (Figure 2A). The systolic ambulatory BP reduction was −24.5 ± 12.8 mmHg in the responders and 3.9 ± 8.3 mmHg in the non-responders (*p* < 0.05). The responders and non-responders were similar with respect to their clinical parameters, except for age. The responders were significantly older patients, with an average age of 66.0 years (Table 1). Next, we focused on the changes in the inflammatory marker levels before and after RDN. Of note, the responders demonstrated significant reductions in hsCRP (4041 (1261–8023) ng/mL and 1909 (499–5110) ng/mL, *p* < 0.05, before and after RDN, respectively) and IL-6 (2.8 (1.5–3.8) pg/mL vs. 1.9 (1.5–2.5) pg/mL, *p* < 0.05, before and after RDN, respectively) concentrations following the treatment (Figure 2B). TNF-α levels were not affected by the treatment.

### 2.5. Effect of RDN on T-Cell Signatures

To analyze the effect of RDN on T-cell signatures, we first investigated the differences between responders and non-responders at baseline. Before RDN, the responders were characterized by significantly higher CD4+ T_EM_ (39 ± 18% vs. 29 ± 12%, *p* < 0.05), lower CD4+ naïve (19 ± 13% vs. 27 ± 7%, *p* < 0.05), and lower CD8+ T_EMRA_ cell frequencies than the non-responders (11 (7–20)% vs. 24 (10–39)%, *p* < 0.05). Furthermore, we analyzed the capacity of CD8+ T_EMRA_ cells to produce pro-inflammatory cytokines upon stimulation in the responders and non-responders. Not only did the non-responders have higher levels of CD8+ T_EMRA_ cells, but they also produced more IFN-γ before RDN (Figure 2D), suggesting that non-responders have a higher capacity of pro-inflammatory T cells, which promotes the chronic low-grade inflammation observed in these patients.

Further, we characterized the T-cell subsets 6 months after the RDN procedure. The responders demonstrated a significant decrease in CD8+ T_CM_ cell frequencies (Table 1), which was more pronounced among the male responders (Suppl. Figure 1). Moreover, the capacity of the CD8+ T_EMRA_ cells to produce the pro-inflammatory cytokines IFN-γ and TNF-α was significantly higher in the non-responders than in the responders (Figure 2D), suggesting that RDN did not affect T-cell function in the non-responders.

As the T-cell response showed differences between the responders and non-responders, we further evaluated the potential predictors of a successful outcome after RDN. Higher CD4+ T_EM_ cell frequencies at study entry were associated with higher systolic and diastolic ambulatory blood pressure reductions (r −0.421, *p* = 0.02 and r −0.413, *p* = 0.02, for systolic and diastolic blood pressure, respectively, Figure 2C). Higher pre-procedural CD8+ T_EM_ cell frequencies were related to a higher ambulatory diastolic blood pressure reduction (r −0.424, *p* = 0.02, Figure 2C), suggesting that higher levels of T effector memory cells are predictors of a successful outcome after RDN.

## 3. Discussion

In the present study, we showed that patients with resistant hypertension (RH) have a more pronounced pro-inflammatory immune response than healthy controls. Furthermore, we demonstrated that non-responders to RDN are characterized by a pro-inflammatory T-cell signature that is unlike that of responders.

Several studies have demonstrated that patients with hypertension show a more pronounced chronic low-grade inflammation than healthy controls [27,28]. Here, we confirmed these results and showed that patients with RH are characterized by higher levels of pro-inflammatory cytokines, such as hsCRP, IL-6 and TNF-α, than healthy controls. In addition to this observation, we found that patients with RH exhibit a pro-inflammatory T-cell signature compared to control subjects, suggesting that, at least in part, a pro-inflammatory T-cell response drives this low-grade inflammation. In detail, the patients with RH had higher frequencies of CD4+ T_EM_ and T_EMRA_ cells, as well as CD8+ T_CM_ cells, than controls. Higher circulating levels of CD4+ memory cells have been described to be associated with senescence [29,30], atherosclerosis and cardiovascular risks [31,32,33,34]. Moreover, higher levels of CD8+ T_CM_ cells have been observed among patients with high cardiovascular risks [35]. These observations may explain why patients with RH have the highest cardiovascular risk [10]. However, since the patients with RH included in this study were older than the healthy controls, we cannot exclude the possibility that these differences in T-cell signatures are, in part, due to older age.

Next, we investigated the effect of RDN on the immune response in the patients with RH. In our cohort, 67% of the patients with RH showed a significant blood pressure reduction 6 months after RDN. These results are in accordance with those of previously published works [15,16,17]. Interestingly, older patients tended to have a more successful response to RDN. Previously published works do not present consistent results with regard to age as a predicting factor for RDN [15,36,37,38]. In general, our study cohort was older than that in other studies. It is difficult to determine to what extent the immunological effects of aging (immunosenescence) or other factors, such as differences in vascular function or the renin–angiotensin system, affect this association, as we did not measure these factors. Among the RDN responders, decreases in hsCRP and IL-6 concentrations following the procedure were observed. Several studies have previously demonstrated reduced inflammatory activity in RDN responders 6 to 12 months after the procedure [29,36,39,40]. These associations suggest that reducing sympathetic nerve activity via RDN might modulate the inflammatory state observed among patients with hypertension. However, it is not clear whether this effect is related to a direct interaction between the SNS and the immune system or to the blood pressure reduction itself, since antihypertensive drug treatment also reduces chronic inflammation in essential hypertension [41,42].

In order to analyze whether the reduced inflammation among the RDN responders was related to a modulated immune cell phenotype and/or function, we further compared the T cytotoxic and helper lymphocyte subsets among the responders and the non-responders. The characterization of immune signatures within the responder group showed that the responders initially had higher CD4+ T_EM_ cell frequencies. In addition to this, higher CD4+ T_EM_ frequencies were associated with a more pronounced decrease in systolic and diastolic blood pressure following RDN. Initially, the RDN responders had lower CD4+ naïve cell frequencies. It should be noted that the significantly higher level of CD4+ naïve T cells in the non-responders in our cohort was also higher than the level among the healthy controls (27±7% vs. 25±11%). This interesting observation suggests that this cell population with a naïve phenotype consists mainly of atypical effector memory cells [43]. These effector memory cells can produce IL-2, TNF-α and IFN-γ as cytokine-producing naive T cells, or memory stem cells. They are likely to rapidly migrate into inflammatory foci and then secrete type 1 cytokines. Unfortunately, we did not further characterize these cells.

Animal studies have suggested an important role of cytotoxic CD8+ T cells in the development of hypertension. Moreover, these T cells seem to be particularly well-activated by SNS overexcitation. [24,26,44,45]. Here, we observed several differences within the CD8+ T-cell subsets among the RDN responders and non-responders before and after RDN, highlighting the interaction between cytotoxic CD8+ T cells, blood pressure control and renal sympathetic nerve activation. First, higher frequencies of CD8+ T_CM_ cells at study entry were associated with higher blood pressure reductions. Second, following RDN, a slight but significant reduction in CD8+ T_CM_ cells among the responders was observed. This reduction might be mainly related to oligoclonal CD8+ T cells participating in hypertension development and persistence [21,26,46]. To determine whether this is the case, additional in-depth CD8+ T-cell phenotyping would need to be performed. Third, the non-responders had significantly higher frequencies of CD8+ T_EMRA_ cells. Interestingly, the CD8+ T_EMRA_ cells of non-responders produced more pro-inflammatory cytokines than those of the responders before and especially after RDN. This very interesting finding could be explained by the fact that norepinephrine binds to different adrenoreceptors expressed on CD8+ T_EMRA_ cells and activates pro-inflammatory cytokine release [24]. Thus, increased norepinephrine release induced by persisting SNS overactivity among the non-responders would lead to an increased release of pro-inflammatory cytokines, as observed in our study [47,48,49]. However, based on this observation and the fact that non-responders are characterized by higher inflammatory markers, it also seems feasible that non-responders to RDN have an overactivated immune response that partially contributes to the difficulty in treating patients with RH [43,50]. Furthermore, one can speculate that an overactivated immune response attenuates the response to RDN, and in this case, the reduction in sympathetic activity would not have a substantial impact on blood pressure. It is noteworthy that we only measured the T-cell subsets in the peripheral blood and not in the target organs. Further studies are necessary to investigate the differences between the abundance and function of activated T cells in the blood and target organs [25]. In any case, it seems feasible that an analysis of immune cell signatures before RDN may provide additional information on the success rates of RDN.

The present study has several strengths and limitations. Although we present results on lymphocyte subsets from patients with known high sympathetic activity, our study population is small, and we used an observational design. Due to ongoing interfering antihypertensive medication, we did not measure aldosterone, renin or norepinephrine during the study. The study lacks an age-matched healthy control group, which would decrease the possibility of biased associations. Furthermore, although the observed changes in the T-cell subsets show a direct association between RDN and T-cell signatures, we cannot exclude that the changes in T-cell subsets are a result of RDN-induced blood pressure reductions. However, the strength of the study is that, for the first time, we demonstrate changes in T-cell subsets following the RDN procedure. Secondly, all patients received 24 h ambulatory blood pressure measurements before and after the treatment, allowing for an objective evaluation of the RDN effect. Moreover, the study was performed in one center, ensuring a strict work-up and follow-up schedule, as well as consistent and uniform pre-analytical sample processing.

To conclude, we were able to confirm the hypothesis derived from animal studies indicating that there is an interplay between sympathetic overactivity and immune response. A specific T-cell signature leads to higher pro-inflammatory activity, which contributes to the persistence of high blood pressure in patients with RH. A thorough analysis of the immune signature might be of advantage to improve patient selection for RDN.

## 4. Materials and Methods

### 4.1. Study Population

A total of 30 patients with resistant hypertension, which was defined according to ESH guidelines, were included in this prospective observational study [8]. The other inclusion criteria were age older than 18 years, no presence of acute infection and no presence of advanced chronic renal failure with an estimated glomerular filtration rate (eGFR) below 30 mL/min/1.73m^2^, and the ability to give informed consent for participation in the study. All the patients with hypertension were on stable antihypertensive treatment for at least 4 weeks and had undergone a diagnostic evaluation for secondary hypertension, which was excluded in all the patients included in the study. In addition to this, 17 healthy controls were included in the study. These subjects had no history of any cardiovascular disease and demonstrated a systolic blood pressure below 130 mmHg without any antihypertensive drugs. This study was approved by the local ethics committee of the Medical Faculty at the Heinrich-Heine University, Düsseldorf, Germany (study number 3848 and 5365R), and it is in line with the Declaration of Helsinki, as revised in 2013.

### 4.2. Renal Denervation Procedure

Bilateral renal denervation (RDN) was performed for the patients with resistant hypertension using a radiofrequency ablation catheter (Symplicity Flex ^®^ or Symplicity Spiral ^®^, Medtronic, Palo Alto, CA, USA) as described previously [51]. Briefly, the ablation catheter was placed via a. femoralis communis and positioned in the renal artery using X-ray guidance. After that, the electrode(s) were heated to about 55–65 degrees Celsius, a temperature high enough to induce nerve fiber damage within the artery wall. In the cases where a Symplicity Flex catheter was used, the catheter was withdrawn 5mm after each ablation to ensure circular ablation within the renal artery, as the catheter uses one radiofrequency electrode. The Symplicity Spiral catheter, however, has a helical shape, and the catheter consists of four electrodes enabling ablation at multiple points simultaneously. The procedure was performed in both renal arteries, and 4–6 points were ablated per renal artery.

### 4.3. Follow-Up of Patients with RH after Renal Denervation Procedure

The patients with resistant hypertension were followed-up for 6 months. Before RDN and 6 months after the procedure, a 24 h ambulatory blood pressure measurement (Mobil-O-Graph NG, Struck Medizintechnik GmbH, Enger Germany), as well as automated office blood pressure measurement (Boso Medicus Vollautomat, Bosch+Sohn GmbH & Co., Jungingen, Germany), was performed, and blood samples were drawn. According to the blood pressure measurements, the patients were divided into two groups: responders and non-responders. The patients with a reduction in systolic automated office blood pressure of at least 10 mmHg or more were defined as responders. 

### 4.4. Measurement of Inflammation Markers

The concentrations of inflammatory markers in patients’ serum were measured at two time points—before and 6 months after RDN. In addition, the same inflammation markers were measured in the 17 healthy controls. The samples were tested for interleukin 6 (IL-6), tumor necrosis factor-α (TNF-α) and high-sensitivity C-reactive protein (hsCRP) using an ELISA kit according to the manufacturer’s instructions (Die Quantikine^®^ Human CRP/TNF-α/IL-6 ELISA Kit, R&D Systems Inc., Minneapolis, MN, USA).

### 4.5. Measurement of T-Cell Signature

The subsets of T cytotoxic (CD8+) and T helper (CD4+) cells were analyzed in the peripheral blood mononuclear cells (PBMCs) of patients with RH before and 6 months after the treatment. Blood samples were drawn using Ficoll (CPT cell preparation tubes, Becton, Dickinson and Company, Franklin Lakes, NJ, USA) tubes. PBMC isolation and freezing were performed as described in the literature [52]. An analysis of the T-cell signatures was performed after all patient samples were collected. For the flowcytometric (FACS) measurements, the frozen PBMCs aliquots were thawed and stained according to recommendations [53]. An antibody cocktail consisting of anti-CD45, anti-CD3, anti-CD4, anti-CD8, anti-CD45RA and anti-CCR7 was added to the thawed PBMCs (Supplementary Table S1). The cell populations of the PBMCs were separated via FSC (forward-angle light scatter) and SSC (side-angle light scatter). Leukocytes were identified using CD45 staining, and T cells were identified using CD3 antibodies. Further gating was performed using CD4 and CD8 antibodies. According to the expressions of the surface markers CD45RA and CCR7, CD4+ and CD8+ cells were divided into T central memory cells (T_CM_ = CD45RA−, CCR7+), T effector memory cells (T_EM_ = CD45RA−, CCR7−), T effector memory cells (T_EMRA_ = CD45RA+, CCR7−) and T naïve cells (T_naïve_ = CD45RA+, CCR7+), as presented in Supplementary Figure S2. According to previously published protocols, intracellular staining was performed [54]. In short, T cells were sorted using the antibodies listed in Supplementary Table S1 utilizing a high-speed digital cell sorter (Beckton Dickinson and Company) and re-stimulated for 4 h at 37 °C, 5% CO_2_ in RPMI-1640 medium (Sigma Aldrich, St. Louis, MI, USA) supplemented with 10% fetal calf serum, 100 U/mL penicillin (Sigma Aldrich), 100 mg/mL streptomycin (Sigma Aldrich), 50 ng/mL PMA (Sigma Aldrich), 250 ng/mL ionomycin (Sigma Aldrich) and 0.65 µL/mL Golgistop (BD, Franklin Lakes, NJ, USA). The restimulated cells were fixed and permeabilized with a Foxp3/Transcription Factor Staining Buffer kit (eBioscience, San Diego, CA, USA) according to the recommendations of the manufacturer. Intracellular cytokines were detected using antibodies against human tumor necrosis factor alfa (TNF-α) and interferon gamma (IFN-γ), as presented in Supplementary Figure S3. An analysis of T-cell subsets was carried out using flow cytometry (CytoFlex^®^, Beckman Coulter, Brea, CA, USA) and the software Kaluza ^®®^ (Beckmann Coulter).

### 4.6. Data Analysis

A statistical analysis was performed using SPSS version 23 (SPSS Inc., Chicago, IL, USA) and Graph Prism 8 (GraphPad Software, San Diego, CA, USA). The type of data distribution was assessed, and the continuous variables are expressed as mean ± standard deviation (SD) or median with the interquartile range expressed as two numbers, Q1–Q3. Categorical variables are expressed as numbers (percentages). The differences between the groups were assessed using unpaired or paired *t*-test, Mann–Whitney test or Wilcoxon rank test where appropriate. Univariate logistic regression was used to indicate variables associated with a positive response to renal denervation. *p* values less than 0.05 were considered statistically significant.

## Figures and Tables

**Table 1:** Characteristics of responders and non-responders before and after the renal denervation procedure.

Parameter	RH		RH Treatment	
	Responder	Non-Responder	Responder	Non-Responder
Age, y	66.0 ± 5.8	51.2 ± 12.2 ***	66.5 ± 5.8	51.7 ± 12.2 ***
Sex (M:F)	12:8	5:5	12:8	5:5
AOBPM, mmHg	169 ± 15/92±18	167 ± 18/92 ± 19	139 ± 14/78 ± 10 ^###^	167 ± 16/89 ± 10 **
ABPM, mmHg	159 ± 17/90 ±12	150 ± 6/90 ± 8	139 ± 14/78 ± 10 ^###^	155 ± 8/85 ± 10 **
Antihypertensive, N	5.0 ± 1.0	6.0 ± 1.0	5.0 ± 2.0	6.0 ± 1.0
Antihypertensive class				
― ACEi/ARB	17 (85%)	10 (100%)	18 (90%)	10 (100%)
― MRA	4 (20%)	2 (20%)	7 (35%)	3 (30%)
― CCB	17 (85%)	8 (80%)	16 (80%)	8 (80%)
― Diuretic	19 (95%)	10 (100%)	15 (75%)	10 (100%)
― β-Blocker	12 (60%)	8 (80%)	14 (70%)	8 (80%)
― α-Blocker	7 (35%)	4 (40%)	6 (30%)	2 (20%)
― Other	17 (85%)	8 (80%)	16 (80%)	7 (70%)
BMI	30.2 ± 5.8	30.8 ± 5.8	30.2 ± 5.8	30.8 ± 5.8
OSAS, N (%)	14 (70%)	4 (40%) *	14 (70%)	4 (40%) *
Diabetes, N (%)	5 (25%)	3 (30%)	5 (25%)	3 (30%)
Hb1AC, %	6.0 ± 0.9	5.9 ± 0.7	6.0 ± 0.9	5.8 ± 0.7
eGFR, mL/min	72 ± 27	75 ± 28	72 ± 27	75 ± 28
hsCRP, ng/mL	4041 (1261–8023)	3063 (1043–8823)	1909 (499–5110) ^#^	2745 (1331–10,099)
TNFα, pg/mL	2.1 (1.5–2.9)	2.6 (1.4–3.7)	2.3 (1.8–2.8)	2.7 (1.7–5.4)
IL-6, pg/mL	2.8 (1.5–3.8)	2.5 (1.7–3.6)	1.9 (1.5–2.5) ^#^	3.2 (2.0–3.7) *
CD4, %	65 ± 17	61 ± 14	65 ±15	63 ± 14
CD4 T_CM_, %	39 ± 17	38 ± 15	36 ± 15	39 ± 16
CD4 T_EM_, %	39 ± 18	29 ± 12 *	38 ± 19	25 ± 8 *
CD4 T_EMRA_, %	4 (2–8)	7 (2–12)	5 (3–9)	4 (2–10)
CD4 naïve, %	19 ± 13	27 ± 7 **	20 ± 15	26 ± 7
CD8, %	35 ± 17	39 ± 14	35 ± 15	37 ± 14
CD8 T_CM_, %	17 ± 8	15 ± 9	14 ± 7 ^#^	17 ± 8
CD8 T_EM_, %	26 ± 13	24 ± 9	25 ± 12	21 ± 8
CD8 T_EMRA_, %	11 (7–20)	24 (10–39) *	11 (7–17)	25 (9–44) **
CD8 naïve, %	38 ± 13	37 ± 17	38 ± 15	33 ± 14
CD4/CD8	2.3 (1.1–4.4)	1.4 (0.9–2.9)	2.2 (1.0–3.5)	1.5 (1.0–3.3)

RH—resistant hypertension, AOBPM—automated office blood pressure measurement, ABPM—ambulatory blood pressure measurement, ACEi/ARB—angiotensin-converting enzyme inhibitor and angiotensin receptor blocker, MRA—mineralocorticoid receptor antagonist, CCB—calcium channel blocker, BMI—body mass index, OSAS—obstructive sleep apnea syndrome, Hb1AC—hemoglobin A1c, eGFR—estimated glomerular filtration rate, hsCRP—high-sensitivity C-reactive protein, TNF-α—tumor necrosis factor alfa, IL-6—interleukin 6, CD4+—helper T cells, CD8+—cytotoxic T cells, T_CM_—central memory cells, T_EM_—effector memory cells, T_EMRA_—effector memory residential cells. * indicates differences between responders and non-responders; # indicates differences between before and after treatment within the responder or non-responder group. * *p* < 0.05, ** *p* < 0.01, *** *p* < 0.001, or ^#^ *p* < 0.05, ^###^ *p* < 0.001 using unpaired or paired *t*-test, Mann–Whitney or Wilcoxon test where applicable.

## Data Availability

Raw statistical data can be provided upon request. Please contact the corresponding author for more information.

## Supplementary Materials

The following supporting information can be downloaded at https://www.mdpi.com/article/10.3390/ijms24032493/s1, Figure S1: Comparison of T-cell subsets among responders with regard to sex. Table S1: Antibodies used for extracellular and intracellular lymphocyte staining and their specifications. Figure S2: Gating strategy on T-cell memory phenotypes. Figure S3: Gating strategy on T-cell effector memory residential cells and their intracellular cytokines.
