# Supplementary material for: The Effect of Renal Denervation on T Cells in Patients with Resistant Hypertension
Source: Int J Mol Sci. 2023 Jan 27;24(3):2493. doi: 10.3390/ijms24032493 (PMC9917284; doi:10.3390/ijms24032493)
Supplement: Supplementary file 1 [file ijms-24-02493-s001.zip › ijms-2150815-supplementary.pdf]

Supplementary Material

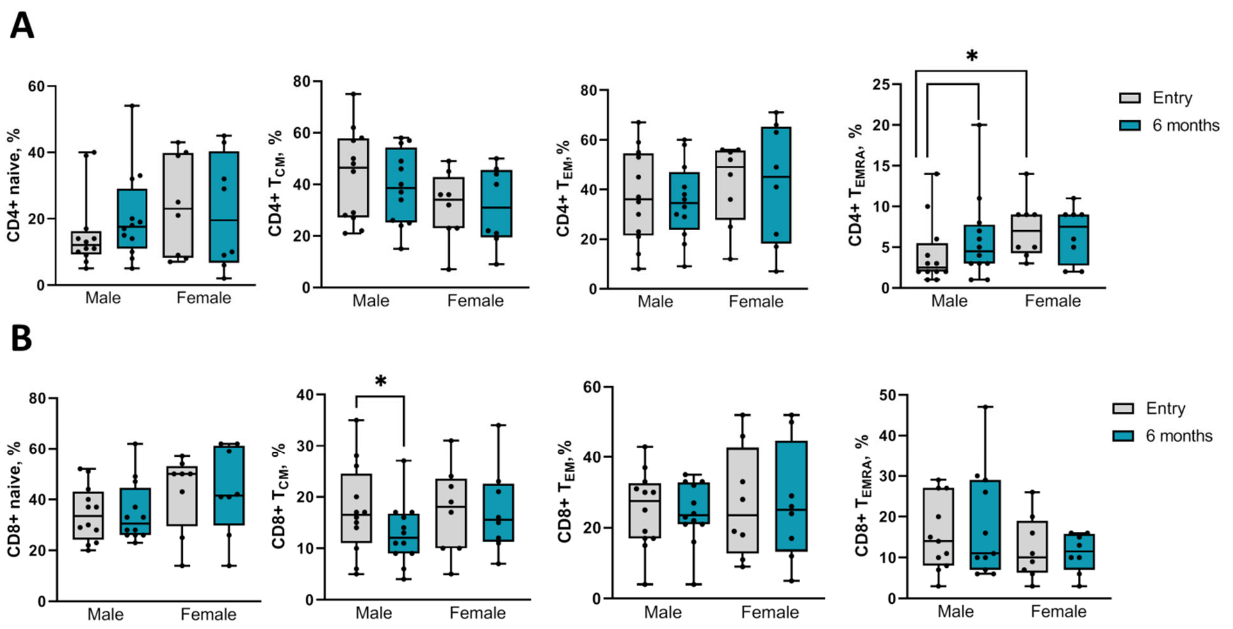

Supplementary Figure S1. Comparison of T cell subsets among responders with regard to gender. A – the frequencies of T helper cells among responders before and after renal denervation procedure. B - the frequencies of T cytotoxic cells among responders before and after renal denervation procedure. CD4+ - T helper cells, CD8+ - T cytotoxic cells, T_{CM} – central memory cells, T_{EM} - effector memory cells, T_{EMRA} – effector memory residential cells. * represent significant difference between the groups with $p < 0.05$ using unpaired t – test or Mann Whitney test.

Supplementary Table S1. Antibodies used for extracellular and intracellular lymphocyte staining and their specifications.

Antibody	Clone	Ig subclass	Conjugate	Species	Manufacturer	Reference number
**Extracellular staining**						
**CD3**	SP34-2	IgG1k	BV786	Mouse	BD Horizon	563918
**CD45**	HI30	IgG1k	BV650	Mouse	BD Horizon	563717
**CD4**	SK3	IgG1k	BV605	Mouse	BD Horizon	565998
**CD8**	SK1	IgG1k	APC A700	Mouse	BD Horizon	565192
**CD45RA**	2H4	IgG1k	PacificBlue	Mouse	Beckman Coulter	A82946
**CCR7**	150503	IgG2a	PECF594	Mouse	BD Horizon	562381
**Intracellular staining**						
**CD3**	UCHT1	IgG1k	PacificBlue	Mouse	Biolegend	300431
**CD8**	OKT-8	IgG2ak	FITC	Mouse	eBioscience	11-0086-42
**CD45RA**	T6D11	IgG2b	PerCP-V700	Mouse	Miltenyi Biotec	130-097-693
**CCR7**	FR11-11E8	IgG1k	PE	Mouse	Miltenyi Biotec	130-093-621
**IFN-γ**	45-15	IgG1k	PE-V770	Mouse	Miltenyi Biotec	130-096-752
**TNF-α**	cA2	IgG1k	APC	Mouse	Miltenyi Biotec	130-117-382

A

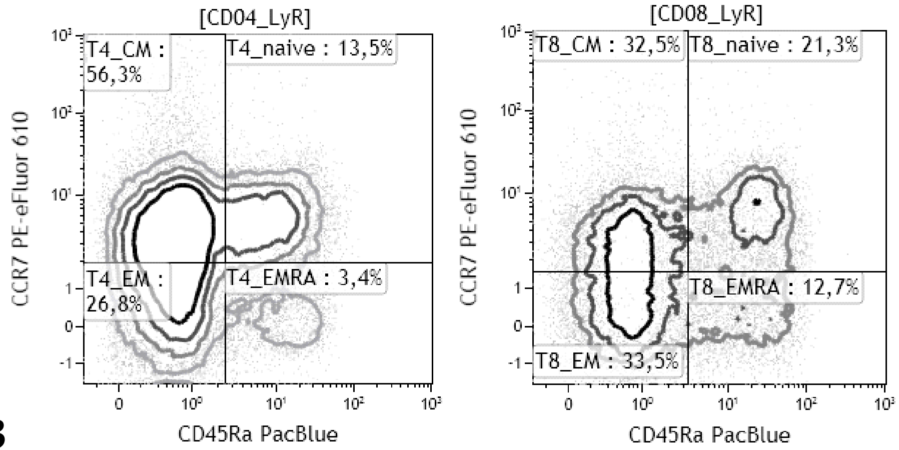

B

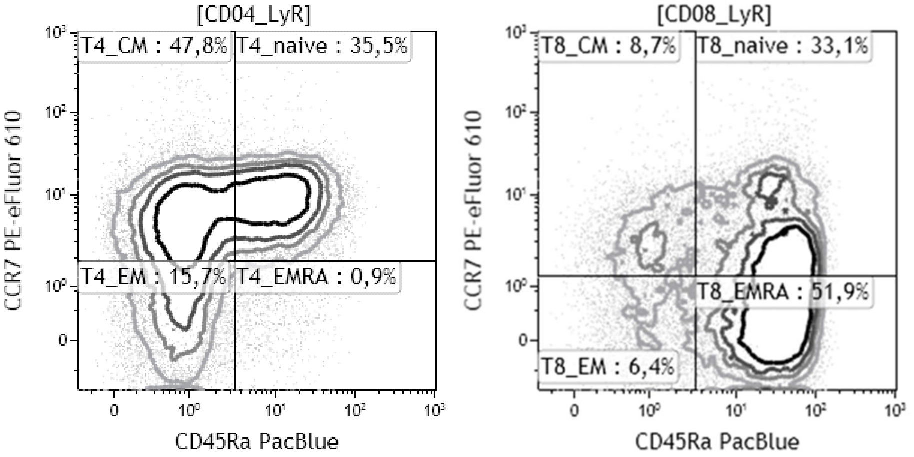

Supplementary Figure S2. Gating strategy on T cell memory phenotypes. After identification of CD4+ and CD8+ positive lymphocytes, the cells were further analyzed for their CCR7 and CD45Ra expression. Accordingly, the cells were divided into 4 groups: T central memory cells (T_{CM}=CD45RA-,CCR7+), T effector memory cells (T_{EM}=CD45RA-,CCR7-), T effector memory residential cells (T_{EMRA}=CD45RA+,CCR7-) and T naïve cells (CD45RA+,CCR7+). A – a representative gating of RDN responder. B - a representative gating of RDN non-responder.

A

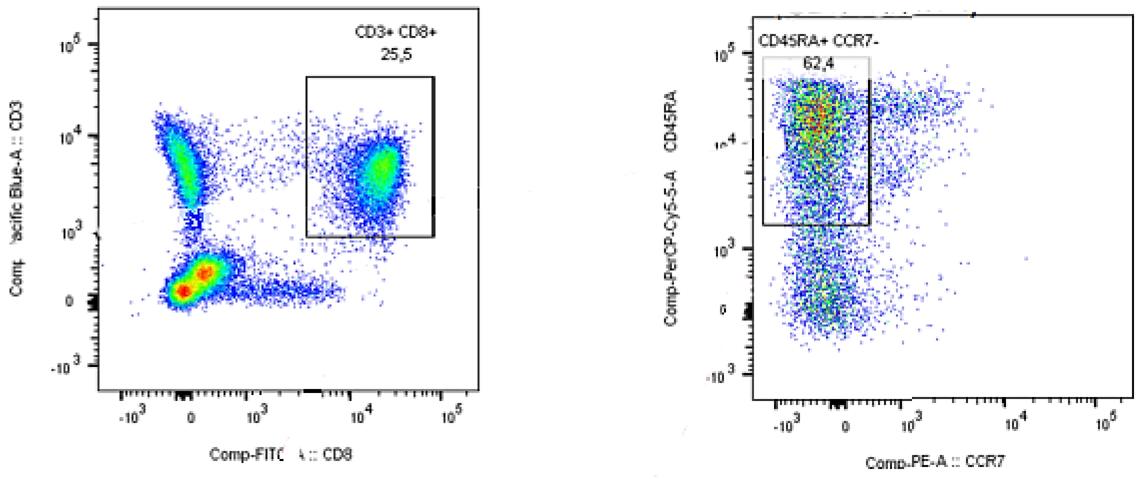

B

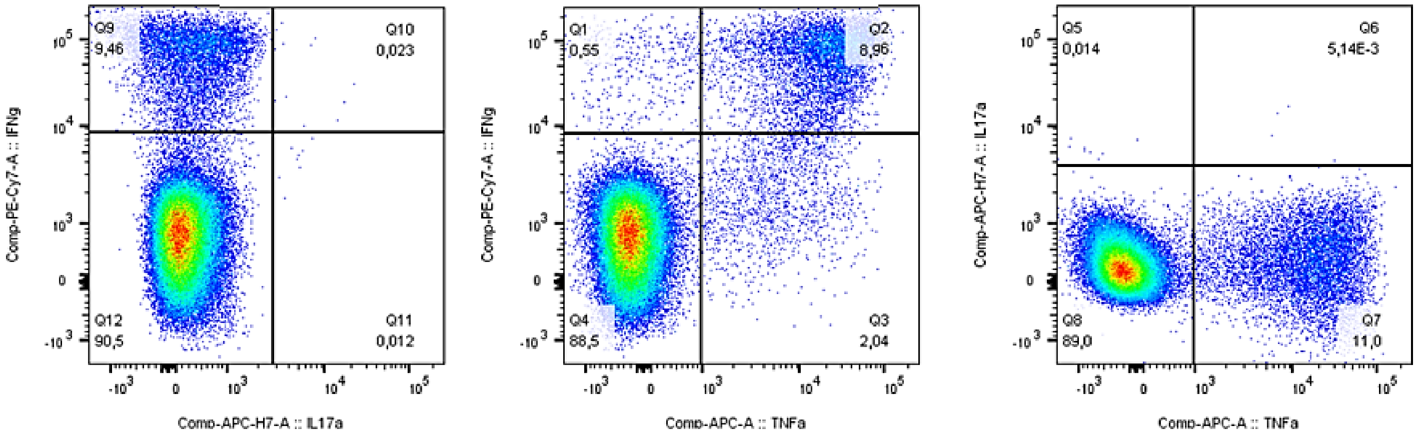

Supplementary Figure S3. Gating strategy on T cell effector memory residential cells and their intracellular cytokines. After identification of CD8+ positive lymphocytes, the cells were stained with anti-CCR7 and anti-CD45Ra. T effector memory cells (TEMRA) were defined as CD45Ra+ and CCR7+ (A). After that, the intracellular cytokines in TEMRA cells were identified using anti-TNF-α and anti-IFN-γ staining (B).
